# Supplementary material for: Verification and reproducible curation of the BioModels repository
Source: PLoS Comput Biol. 2025 Dec 4;21(12):e1013239. doi: 10.1371/journal.pcbi.1013239 (PMC12677764; doi:10.1371/journal.pcbi.1013239)
Supplement: Attachment — Submitted filename: Response to Reviewers.pdf [file pcbi.1013239.s001.pdf]

Response to Reviewers:

A previous submission of this work received the following review from an editor:

(from "Decision: PCOMPBIOL-D-25-00074 - [EMID:d767eac528870a0a]")

"*It looks an interesting study. However, the current version is too short and the key details of the methods and unique contributions are missing, thus, we cannot make a fair decision based on the current version. A more detailed version with expanded information would be appreciated for re-consideration.*"

As requested, the new version of the paper has been greatly expanded, from nine and a half pages to thirteen. Every section has received major updates, these include:

- The Introduction section now better sets up the state of the art in the field, the motivation, and how our work contributes to and extends that.
- The Methods section was (as requested) expanded to include many more details, including a new figure illustrating a typical issue that was addressed.
- The Results and Discussion section was expanded to emphasize how specifically this work contributes to the field.

Thank you for your consideration.

Lucian Smith
