# Supplementary material for: Verification and reproducible curation of the BioModels repository
Source: PLoS Comput Biol. 2025 Dec 4;21(12):e1013239. doi: 10.1371/journal.pcbi.1013239 (PMC12677764; doi:10.1371/journal.pcbi.1013239)
Supplement: Attachment — Submitted filename: Response_to_Reviewers_auresp_1.pdf [file pcbi.1013239.s002.pdf]

We would like to thank all three reviewers for their comments. We have addressed all of them in the new version of the paper, which is now longer but also significantly more robust. The major changes include:

- A detailed discussion and definitions of different levels of reproducibility.
- More history of SBML reproducibility, including a discussion of the SBML Test Suite, and an earlier analysis of BioModels.
- The 'secondary file updates' section was reduced and moved to later in the paper.
- A new BioModels curation protocol figure was added.
- More details about fixes were included.
- A significant section was added that addresses the remaining discrepancies, and what would need to be done to fix them. This includes a new figure showing results from two BioModels where all five simulator results failed to match, but for which some overall similarities can be clearly seen.
- A discussion of other modeling paradigms that could be addressed by a similar project was added.

Other changes were made throughout the paper to improve clarity. Particular bits of new text are added in red after the comments, though for reviewers 2 and 3, the changed sections were already quoted in responses to reviewer 1. Thanks again to the three of you for your insightful comments.

Reviewer #1:

This manuscript from Smith and others claims to have generated a revised Biomodels database collection that contains verified and reproducible models, whereas before the database contained many models that were not so. The primary definition they use is that each model in SBML form should be able to be paired with a SED-ML document for specifying simulations, and then simulated by at least two different tools to obtain the same time course information. A main issue is that this definition of a reproducible model seems to fall short of what many would consider to be, is that the model in the database reproduces the original claims of the model and/or simulation data given in the original publication. The fact that an SBML model and a SED-ML paired document might give different results using different simulators seems much more likely due to the simulator having issues, and/or the wrappers that are used to convert the information to the format needed for the simulator. In fact, in one of the few author's examples (Fig. 3), this is the case. Thus, the manuscript as written does not seem to support the very broad claims being made. That being said, the set of wrappers written to interface a common SBML+SED-ML file set to a variety of simulation tools could be a useful contribution to the modeling community, and that in this reviewer's opinion is one of the novelties of this work. However the fidelity of these wrappers is uncertain, given the extremely surprising lack of agreement between many simulators, which seem to all be using standard ODE solvers. Some more focus on why so much disagreement is seen would be very important and is quite disturbing.

Thank you for these insights! It was not our intent to claim that as a result of this work, all ODE models in the BioModels database are fully reproducible with respect to the original publications. Rather, they are now more reproducible than they were before. A new section has been added to the 'Summary of Results' detailing the different levels of reproducibility that existed before our work started, and where things stand now as a result of our improvements.

We agree that insufficient time was spent analyzing the causes of the remaining discrepancies. New material has been added to the Conclusion section outlining the general state of where the remaining discrepancies likely come from. Hopefully this helps illustrate why our results were not at all surprising to us, and matched exactly what we've seen over the preceding decade: many computational results that are not even repeatable on the same architectures as originally produced the results, let alone reproducible in new contexts. We appreciate this perspective, as it reminds us that naturally, one *should* expect results to be repeatable across simulators, and the fact that they do not is a serious breach of the trust simulators promise to their users. One's choice of simulator should be a matter of preference and convenience to the user, not one that will materially affect simulation results. That is indeed the world we are striving towards, and our hope is that this work and this paper will push the field closer to that state. But we absolutely have not arrived there yet.

"Determining the scope of what remains to be done is hard to discern, but the general shape can at least be roughly determined. Many issues are already known: models that failed to run with a particular wrapper (845 runs altogether, compared to the 4430 successes) all have error messages that can be examined. Determining the problem with runs that failed to match any other simulator is more complicated, as there is no obvious error message to start with. Here, there are 1624 pairwise comparisons that failed, compared to 6016 comparisons that succeeded. (Remember that the more simulators that successfully produce output, the more possible pairwise comparisons there are: when all five produced output, 10 comparisons are possible.)

In many cases, the model in question contains a feature of SBML that is not supported by the simulator. For example, Amici does not support delayed events (used in 56 BioModels), neither Amici, PySCeS, nor VCell support assigned stoichiometries (used in two BioModels), and VCell does not support non-integer stoichiometries (used in 33 BioModels). Considering solely the SBML features tested in the SBML Test Suite, this explains 174 of the 845 runs that failed (21%), plus an additional 92 of the 1624 failed pairwise comparisons (5.7%), for cases where a simulator is known to not handle certain inputs, but still produces output. As a specific example, there are 7 models in BioModels that use delay differential equations. None of our five tested simulators support this construct, but Copasi and PySCeS produce output anyway. These outputs do not match each other, and they certainly do not match the modeler's intent.

Other failures are due to the SED-ML using features not supported by the wrapper. The VCell wrapper, for example, does not support repeated tasks or steady state analyses (91 of the 'Full' SED-ML models), and other wrappers similarly supported only a subset of those analyses. This explains why the 'template' SED-ML had better success rates than the 'full' SED-ML: the latter was more likely to use uncommon features of SED-ML that our wrappers did not universally support.

For some comparisons, our criteria may have simply been too strict: the magnitude of the variables involved and the scope of the model itself might have warranted a less stringent tolerances. Of the 1624 failed pairwise comparisons, 378 of them (23%) result in a match score between 1 and 10, where '1' is the maximum cutoff for whether two data sets match or not. These models are good candidates for further examination, since slightly different tolerances could have resulted in a match. Each such comparison would need to be examined by hand to determine whether this was the case or not, but false negatives that can be examined by hand are much easier to find than false positives that seem to match but actually have hidden significant differences.

While a complete analysis of the remaining failures and mismatches will take time, we did a 'spot check' of the 21 models which every simulator successfully produced results for, but no two models

matched. For thirteen of these, all five visually looked ‘the same’, even though their match scores were too high. For the remaining eight, at least two of them visually matched, but there were obvious mismatches with the rest. Over the whole set, half of them had oscillations or other rapid changes in values, which could in theory explain some of the results that looked the same but scored poorly: very slight phase differences between results could end up with significantly different numerical values. For oscillating output, therefore, a different method of comparison may be necessary. Figure 6 shows two sets of results where no pair of results passed our ‘match’ criteria. For the first (A) from BioModel 19 [35], Amici and VCell’s results seem to be close to each other, but different from the others, and Copasi and Tellurium’s results are similarly close to each other, but different from the rest. For the second (B) from BioModel 117 [36], all five are close, but contain the rapid oscillations that may have prevented them from passing our ‘match’ test. Determining which results from BioModel 19 are intended, and which represents the most accurate interpretation of the SBML model they all purport to simulate will take further research. Similarly, discerning whether the small differences from the BioModel 117 results (expanded in Figure 6C) are significant or trivial will necessitate revisiting the original paper.

But whether or not the failures can be categorized, it is simply often the case that hidden problems are only revealed when something is explicitly tested. A major contribution of this work is simply the testing framework it provides, for both the simulators we tested and the simulators we did not.

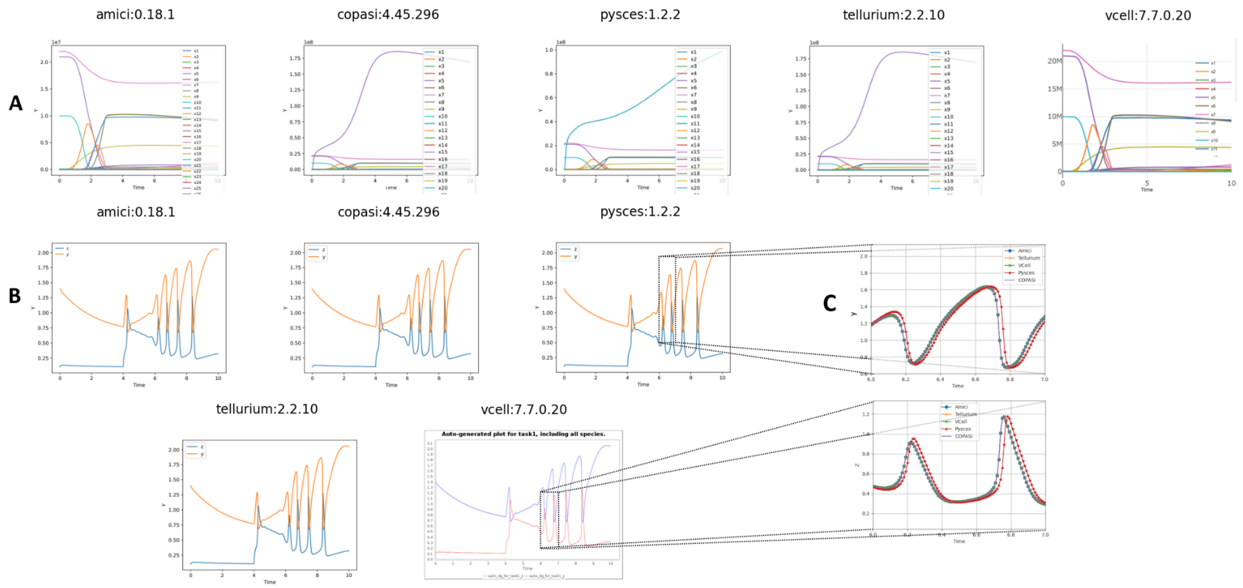

Fig 6. Simulation results from BioModel 19 (A) [35] and from BioModel (B) [36], from all five wrapped simulators. The two graphs in C show a zoomed-in subset of the data from BioModel 117: all five simulator results on a single graph for y (top) and z (bottom). No pair of simulator results passed our ’match’ threshold, for reasons that are obvious to the eye for Biomodel 19, but for reasons that can only be seen at larger scales for BioModel 117. "

- *If separate simulation engines do not produce the same result, it may not be the issue of the model but could be an issue with the software, or a numerical methods issue. Therefore, this criteria may be too aggressive. In fact, the result highlighted in Figure 3 suggests this is the case. Why would someone want to simulate a model with multiple ODE solvers to prove reproducibility?*

There is no guarantee that the simulator first used by an author does not have some idiosyncratic behavior that is not generally accepted as correct by other simulation software. Therefore, *repeatability* (running the same software and getting the same results) is not as strong a signal of scientific robustness as *reproducibility*, when new software is used that utilizes the same underlying scientific reasoning: it ensures that the conclusions are based on known scientific reasoning rather than potentially idiosyncratic behavior. A definition of 'repeatability' and the difference between it and reproducibility has been added to the introduction.

"We define *repeatability* to mean that the computational experiment may be repeated using the same software and computational environment. Although important, repeatability is a much weaker signal of scientific robustness, as unknown errors within the single software environment may be responsible for the reported conclusions."

- *Reference to some of the debate that went on with regards to metabolic modeling and discussion in the introduction would be quite helpful*
    - *o 10.15252/msb.20156548*
    - *o 10.1038/ncomms5893*
    - *o 10.15252/msb.20156157*

Thank you for these references! They do indeed tell a parallel story to our own, in the realm of FBC modeling with SBML. A mention of this has been added.

"Similar comparisons with FBC models across simulators [38] have spurred debates [39, 40] regarding defining the standards and solver capabilities that together determine whether results from a flux balance model analysis can be reproduced."

- *The introduction is generic and more detail on what problems reproducibility of models brings, and how people have already tried to solve it, would be appropriate.*

We have added more about the history of reproducibility to the introduction in the 'SBML' section.

"To encourage SBML simulators to interpret models consistently, the SBML Test Suite [13] was developed starting in 2003 so all simulators would have a common set of unit tests that checked different aspects of the SBML specification, organized by labeled ‘tags’, so that when a particular simulator doesn’t support a particular SBML feature algebraic rules, fast reactions, etc.) they can flag the tests marked with that tag as not supported. Voluntarily, simulator authors may make their results public in the SBML Test Suite Database [14].

In 2008, Bergmann and Sauro [15] took a different approach towards simulator comparisons by publishing a comparison of simulator results of ten-second simulations of all 150 (at the time) curated BioModels. The differences that this work uncovered spurred discussion and activity, as simulator authors discussed the proper interpretation of certain SBML constructs, and changed their own simulators accordingly. Unfortunately, the infrastructure that allowed those comparisons only lasted through 2011, but the changes it promoted survived in the updated simulators."

- *The introduction conflates to some extent open source (accessible) versus reproducibility; this distinction deserves some nuance.*

The introduction now contains much more detail on precisely what is meant by reproducibility, so this distinction is now hopefully more clear. The terms 'open source' and 'accessible' do not appear in the introduction at all.

- *A figure which shows the EMBL protocol for model curation, versus the improvements in this work, would be helpful to the reader.*

We have added a new figure outlining the EMBL's curation procedure, augmented with the new steps this work made possible.

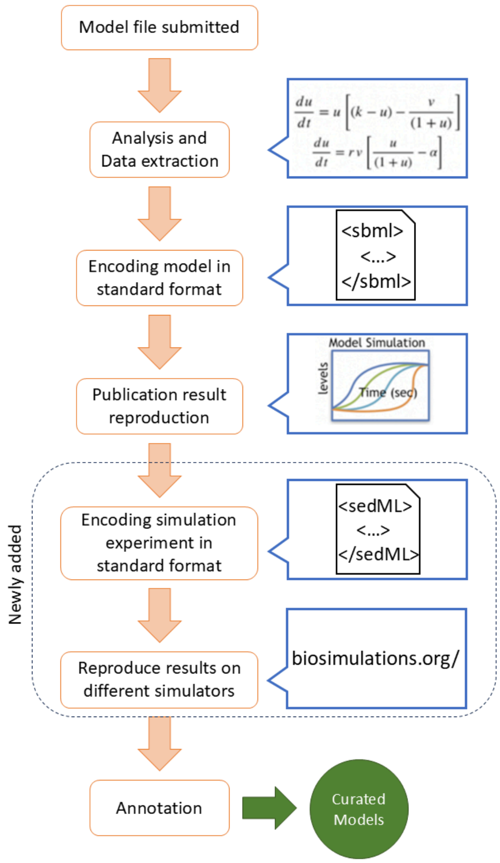

- *In the introduction, the section on BioSimulators is informative but how it is relevant to the described work and how it is used could be better described.*

Clarification of how BioSimulators were used has been added to the paper, under the "BioSimulators" section.

- *The possibility that the developed wrapper codes to enable SED-ML to be interpreted by different solvers play a role in observed errors was not adequately considered. That being said the development of these wrappers seems to be a large part of the novelty of this work but the way the manuscript is presented does not capture this very well.*

We apologize for not making this clear: we agree that the wrappers can and have played a role in the observed discrepancies between simulators; the conclusion section has been modified to emphasize this more.

"Other failures are due to the SED-ML using features not supported by the wrapper. The VCell wrapper, for example, does not support repeated tasks or steady state analyses (91 of the 'Full' SED-ML models), and other wrappers similarly supported only a subset of those analyses. This

explains why the ‘template’ SED-ML had better success rates than the ‘full’ SED-ML: the latter was more likely to use uncommon features of SED-ML that our wrappers did not universally support."

- *Line 185, the paragraph about SED-ML updates, was unclear. If the update happened back in 2021, why is it relevant to this 2025 paper?*

You are correct; those particular updates can be classified as having occurred before the bulk of this work; the paragraph in question has simply been removed.

- *Line 210, what problems, and how would they be fixed? More broadly in this section, how do secondary files fit into the main point of the manuscript of computational reproducibility? Related, Line 214, it was unclear what translation programs did or why they were important.*

On reflection, we decided that this part of the work, while important to have carried out, is probably less important to the message of this paper. The section has been reduced to a briefer summary and moved to later in the paper. An explanation of why this is important (even though it does not directly affect reproducibility) was also added.

"As one example, many BioModels included a PDF file that summarized the contents of the SBML in a human-readable form. This file is not strictly necessary for reproducibility, but as a basic quality control measure, the contents of the PDF should actually match the contents of the SBML file it is supposed to be summarizing."

- *The usefulness of the template SED-ML files for 579 entries with respect to reproducibility of a model for the reported results was unclear, but this seems to be the major point of the paper. More impactful would be SED-ML files that proved reproducibility of results.*

It is absolutely true that SED-ML files designed to actually produce a published figure would be more useful than our generic 'run for 10 seconds' files! However, there is no automated way to produce these files from the information present, and thus will need to be individually produced by hand in the future. A new section defining levels of reproducibility has been added, with the 'results' section updated to emphasize this fact.

"This improved the reproducibility of the curated BioModels. Originally, the models were what might be termed ‘historically reproduced’: at least one curator had been able to use the model to reproduce a figure in the paper, using a different simulator than had been used in the original study. However, working instructions on how a user might reproduce the curator’s work was not able to be stored. Still, a user of the SBML file could be assured that it had been used to reproduce a figure at least once.

The addition of a ‘template’ SED-ML file made models additionally ‘functionally reproducible’: any user could now test different simulators to see which ones can both handle model and produce results in line with other simulators. Even though these SED-ML files do not describe how to reproduce a figure from their original publication, they do provide a common template to check simulators against each other. This work mirrors a similar study in 2008 [15] where simulations of BioModels were compared, and numerous differences between simulators were brought to light and subsequently fixed.

When SED-ML that describes the actual published experiment is available, the experiment it describes has ‘end-to-end reproducibility’: anyone with a compatible simulator can use it to follow the same procedures and obtain compatible results. Those results can then be compared by eye with the original published figures, and compared numerically across simulation platforms.

If not only figures but numeric results are available, a paper may have ‘data-backed reproducibility’: not only can the procedures be replicated in new environments, but the results can be compared with the actual values from the original study. Unfortunately, these numbers are rarely available, and researchers who wish to reproduce those results must instead rely on eyeballing the published figures, or back-calculating approximate values from pixel positions. As such, our efforts here were limited, at this time, to ‘functional’ and ‘end-to-end’ reproducibility. These reproducibility levels are detailed in Table 1, and illustrate the increased functionality that each level provides."

- *It was unclear how many of the 1055 models are reproducible in terms of the claims and/or results of the publications.*

The results section has been updated to emphasize that 546 models with 'template' SED-ML were validated to match on multiple simulators (dubbed 'functionally reproducible'), and an additional 386 'full' SED-ML models were validated to match on multiple simulators (dubbed 'end-to-end reproducible'). Overall, this was 932/1055 models, or 88%.

- *Why was no attention paid to the non-ODE models?*

For this project, our scope was the curated branch of the BioModels database, which only has a handful of non-ODE models. A discussion of what a similar project targeting other modeling paradigms would necessarily entail has been added to the 'future work' section.

"This approach can also be extended to modeling paradigms besides ODEs: SBML itself has the ability to store and standardize different models like flux balance constraint (FBC), spatial, and qualitative models. In addition, SED-ML is designed to be flexible enough to store computational experiments for those model types, and multiple simulators exist that can perform those experiments and whose results can then be compared. Thus, if instructions for experiments can be encoded in a standard way for standardized models (perhaps using SED-ML and SBML), they could be packaged in OMEX files, sent to different simulators, and their outputs compared. The most difficult part of this process will be bridging the gap between the instructions and the simulators: native support for SED-ML could be added to the simulators themselves, or new wrappers could be developed that translated those instructions, as in this study. Efforts along these lines have already started, with the introduction of the FROG analysis for FBC models [37], in which the model’s ‘Flux variability, Reaction deletion, Objective function, and Gene deletion’ are tested. As of yet, no harness exists to automatically produce the desired outputs, but the instructions, at least, are standardized. Similar comparisons with FBC models across simulators [38] have spurred debates [39, 40] regarding defining the standards and solver capabilities that together determine whether results from a flux balance model analysis can be reproduced."

- *Figure 4 is hard to interpret--why are there six different bars but also five different colors in each bar? One wonders as well what is the significance of the template versus full sed-ml comparison. Lastly, there seems to be an unexpectedly large amount of mismatch if the figure is being interpreted correctly, which is very surprising and deserves more analysis. This figure would benefit from changing and/or a much more detailed legend.*

The caption and discussion of Figure 4 has been updated to answer these questions: the six bars were for models that ran on 0-5 simulators, and the colors were for models that matched on 0, 2, 3, 4, or 5 simulators (as it was impossible to 'match' a single simulator). The template vs. full comparison ties into the 'levels of reproducibility' that we had not been explicit enough in our discussions for the first version of the paper, but has now been expanded. The new section in the conclusions discussing known and unknown sources of error also hopefully addresses this point.

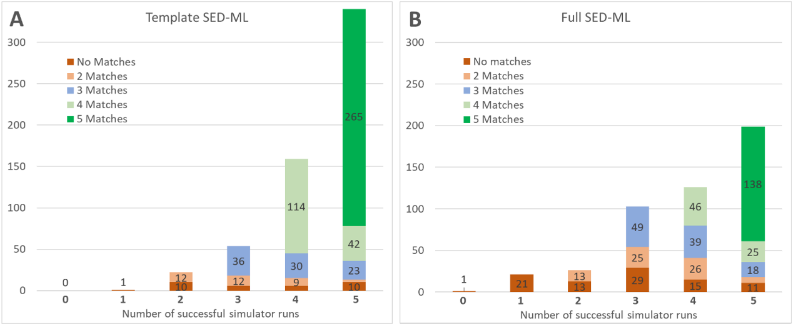

- *Line 381 paragraph, this work that went into changing and updating simulators seems perhaps significant but is not deeply described or enumerated in the paper. More of this would be relevant in this reviewer's opinion.*

While a full list of bug fixes would be tedious, a sampling of a few of them are now included in a new Table 3, just to give the reader a sense of the sorts of changes that were needed for this project. We also now point people to the repositories where full lists of all the changes could be found.

"Table 3 shows a handful of fixes and enhancements to some of the software packages we've been working with, though it is by no means an exhaustive list. Full lists of changes can be found in the commit histories of the individual projects."

Table 3. Example Software Fixes and Enhancements

Software	Fixes and Enhancements
Tellurium wrapper	• Convert local parameters to global parameters so they can be changed by Tellurium's native interface.<br>• Fix bug with referenced values in nested repeated tasks.<br>• Handle SED-ML output start times different from initial times.
PySCeS wrapper	• Correctly output variables controlled by rules.<br>• Convert initial assignments to values before handing model to PySCeS (which doesn't support initial assignments).
VCell	• Allow import of models with reserved words as ids.
Copasi	• Fix handling of reactions with parentheses in their names.

- *The fact that this study does not compare simulation results to original results undermines the claim of general reproducibility, and some of the argued significance of the work. This seems to be driven by a strict reliance on SED-ML as a component of reproducibility, which could be overly demanding. How the Biomodels "increase in reproducibility has in*

turn increased the value of the repository to modelers going forward" (line 478) was not completely convincing.

We believe the new section defining levels of reproducibility (along with Table 1, which summarizes them) hopefully addresses this very important issue. We had not intended to present this work as a full solution to every kind of reproducibility, merely that it significantly increased the reproducibility of the models in the repository. We are not aware of any system or standard besides SED-ML that would allow us to accomplish the goal of general reproducibility. Additionally, as we now note, full reproducibility will remain out of reach as long as modelers do not publish full numeric results with their papers, leaving the comparison of results to humans eyeballing figures for general fidelity, without any actual numbers backing this up. Still, these results give modelers the confidence that the simulation is robust across multiple simulators, and are therefore the result of the general theory of ODE modeling, and not reliant on quirks of particular software packages.

- *Table 1 has perhaps one of the most surprising results that a large number of simulators are different. Given these are using a standard ODE solver, this is alarming, but the reasons why are unclear.*

Again, this was not surprising to us at all, but you rightfully point out that this needed more discussion. The new summary of remaining errors includes a discussion of what specifically makes particular simulators fail.

"In many cases, the model in question contains a feature of SBML that is not supported by the simulator. For example, Amici does not support delayed events (used in 56 BioModels), neither Amici, PySCeS, nor VCell support assigned stoichiometries (used in two BioModels), and VCell does not support non-integer stoichiometries (used in 33 BioModels). Considering solely the SBML features tested in the SBML Test Suite, this explains 174 of the 845 runs that failed (21%), plus an additional 92 of the 1624 failed pairwise comparisons (5.7%), for cases where a simulator is known to not handle certain inputs, but still produces output. As a specific example, there are 7 models in BioModels that use delay differential equations. None of our five tested simulators support this construct, but Copasi and PySCeS produce output anyway. These outputs do not match each other, and they certainly do not match the modeler's intent."

Reviewer #2:

I found the terminology usage around "validation" somewhat inconsistent. In the BioSimulators paragraph, the term appears to refer to validating the SED-ML/SBML format, which differs from your earlier definition where validation involves determining the biological accuracy of a model. While I appreciate your explicit definitions of terms in the Verification of Models paragraph, I believe you should also have provided a definition of 'translation' as its meaning was unclear in the context of 'translation programs'.

Thank you for this input! 'validating' has been changed to 'checking' in the BioSimulators paragraph, and the discussion of 'invalid' files has been changed to 'incorrect files'. The discussion of 'translation programs' now just discusses 'programs' that auto-generate files, to prevent further confusion.

The verification results are particularly interesting. It would have been valuable to include a similar analysis using the SBML test-suite, which is widely used by simulation engine developers to verify

their implementations. Your results highlight persistent issues with different applications correctly simulating SBML. The fact that very few models verified across all five simulation packages indicates significant work remains to be done—as you rightly acknowledge. I anticipate that extending your template SED-ML files will likely reveal additional issues.

Because of this comment, I wrote a program to generate new versions of the SBML Test Suite OMEX files (the old OMEX generator was years out of date), and then ran them through our gauntlet of five simulators. In the end, though, the results were not very surprising, as they mostly matched the self-reported results authors of those simulators have published before in the SBML Test Suite Database (https://sbml.bioquant.uni-heidelberg.de/Simulator). It was definitely a useful exercise, as they pointed out a few issues with our wrappers (particularly with 'comp' SBML models), and we plan to individually contact the simulator authors with these results, to ensure that they match their own internal tests. In the end, though, the work seemed somewhat out of scope for the rest of this paper (which has already grown in length and complexity), so we deemed it better to find a different outlet for that work.

However, you are correct that the SBML Test Suite is directly relevant to the goals of this paper, and is now mentioned in the introduction, and used as a point of comparison in the new section speculating about the causes of the remaining observed discrepancies. And as for your other points, we appreciate the encouragement, and equally hope that more issues will be discovered and fixed as a result, and as we and others extend the SED-ML to match actual figures from the papers.

- *The curation of BioModels has historically involved broader collaboration than just the EBI. Initially, researchers from the University of Hertfordshire and Caltech were also involved.*

This is an excellent point, and the corresponding section has been updated accordingly.

- *Regarding standards chronology, SED-ML was not strictly the second standard to emerge, as SBGN predates it.*

Another good point; the text has been updated to not imply that SED-ML came second, merely that it emerged after SBML.

- *The sentence on line 83, page 4 ("However, many such simulators...") appears redundant and could be removed.*

Agreed; fixed.

- *On line 373, page 11, there is a repetition of the word 'only' that should be corrected.*

Fixed.

Reviewer #3:

*This manuscript represents a valuable contribution to model reproducibility and reuse. The authors looked at *all* curated ODE models in the BioModels database (1,055 models) and generated a SED-ML description of a simulation protocol for each. They then ran simulations using five different ODE solvers/simulation engines, namely COPASI, Tellurium, VCell, PySCeS, and Amici. Sometimes, this failed. And when there was run success there was sometimes a consistency*

problem. The authors tried to fix these problems. After this effort, they were able to get a 98% run success rate (1,031/1,055) and a “verification success” flag for 928 or 932 models (88%). Verification success was declared when two or more simulators (given the same simulation instructions) generated indistinguishable results. This report demonstrates some of the challenges of reproducing modeling/simulation results. I think it has major strengths, which are listed below. I think this report will be of interest to many in the biological modeling community. The weaknesses/limitations of the study that I note below do not require major manuscript revisions. Mostly, these points should just be appropriately recognized in the Discussion section.

- *There was verification failure for 12% of the models, even after curation. The causes are discussed (on ~2 pages) but perhaps the analysis could go further? Could the authors say more about why they think so many models have this problem?*

Agreed. A new section has been added that analyzes both why some simulators failed entirely, as well as why some simulators produced different results from each other.

- *In this study, there was a focus on ODE models. There are other types of models. Could the authors say more about how their efforts might be extended to encompass other model types? What challenges would have to be overcome to consider logic/Boolean models, PDEs, agent-based models, etc.?*

A section on what would be necessary to replicate this work for other modeling paradigms has been added to the 'future work' section.

- *The “template” SED-ML files provided by the authors are not for reproducing published figures. This is a disappointment, but it’s understandable. Could the authors say more about why they did not go further? What are the challenges to reproducing results in figures? How could these be overcome? Can model developers do more to promote reproducibility?*

The only reason we did not go further is time: every 'template' SED-ML file has the potential to be upgraded to a 'full' one, but each will need to be processed by hand, essentially re-curating the model. If model developers were to provide SED-ML from the get-go, the whole process would be done! We added an exhortation to do just that to the end of the paper, and emphasized the 'individual curation' aspect of the rest of the potential work to come, and now reference a Xu and Smith paper where we have begun doing exactly this.

- *The manuscript does not spell out the audit trail for fixes. Could the authors better explain the extent of logging and how other researchers can access available documentation of fixes?*

Many of the fixes took place in separate projects, but all were under source control, mostly at github. Certainly the bulk of the changes for this project in particular can be found in the 'temp-biomodels' github project. We now note this in the text, and provide a table (Table 3, above) containing some examples.

- *There was no attempt to verify whether models capture underlying biology. We really want that type of effort, right? How can we get there? Could the authors say more about this point?*

Validation is indeed the 'next step' of this process, and we hope to be able to focus our efforts in this direction in our upcoming projects. Validation has been beyond the scope of the curation process entirely, but it could be added going forward: experimental results could be stored in the OMEX file with everything else, along with instructions (perhaps in SED-ML) for comparing that data with computational results, allowing fully-reproducible VVUQ analysis; something currently not possible. A brief discussion of this has been added to the end of the paper.

- *Success results are reported inconsistently, 932/1055 in some places, and 928/1055 elsewhere. This inconsistency should be fixed.*

Excellent catch; fixed. The correct value was 932.

- *Verification success rate was 94% for 578 “template” protocols but only 85% for 453 “full” protocols. Are more complex SED-ML files a problem? The simulators evaluated don’t fully support SED-ML features?*

In general, more discussion of where exactly the remaining errors came from was missing from the original version of this paper, and we have added a new section addressing this in the new version. To answer this question specifically, yes, the 'full' protocols more often contained experimental design instructions not supported by all of our wrappers.
